# Bioinformatics and Machine Learning–Based Identification of Critical Biomarkers and Immune Infiltration in Venous Thromboembolism

**DOI:** 10.1155/ianc/2202321

**Published:** 2024-11-22

**Authors:** Yajing Li, Hongru Deng

**Affiliations:** Department of Vascular Surgery, Fu Xing Hospital, Capital Medical University (FXH-CMU), Beijing 100038, China

## Abstract

**Objective:** This study aims to use bioinformatics and machine learning algorithms to screen and analyze the key genes involved in venous thromboembolism (VTE) and explore the relationship between these biomarkers and immune cell infiltration.

**Methods:** The gene expression profile with the identifier GSE19151 was downloaded from the GEO database. Differential expression analysis using the limma package was conducted to identify genes that were differentially expressed between VTE and normal samples. Biological activities of these genes were then investigated through GO analysis utilizing the R language package. KEGG and GSEA were also performed to identify key signaling pathways. Furthermore, machine learning techniques were employed to determine hub gene signatures related to VTE, and ROC curves were used to validate the findings. To compare the immune infiltration of healthy and VTE samples, single sample gene set enrichment analysis (ssGSEA) was applied. Lastly, the Spearman correlation coefficient was used to assess the relationship between the expression of hub genes and immune cell infiltration.

**Results:** A total of 628 differentially expressed genes (DEGs) were discovered between the VTE samples and normal samples. GO analysis identified protein polyubiquitination, lysosomal lumen acidification, organellar ribosome, mitochondrial ribosome, ammonium transmembrane transporter activity, and immunoglobulin binding as the processes with the highest abundance of DEGs. KEGG pathway analysis revealed that DEGs were enriched in ribosome, COVID-19, viral infection, oxidative phosphorylation, Parkinson's disease, nonalcoholic fatty liver disease, apoptosis, and cancer. The most prominent KEGG pathways associated with VTE were ribosome, Parkinson's disease, oxidative phosphorylation, Alzheimer's disease, and Huntington's disease according to GSEA findings. DLST and LSP1 were identified as hub gene signatures in VTE by machine learning integrative analysis, and ROC curves confirmed their diagnostic value. Results from ssGSEA indicated a significant difference in the degree of immune cell infiltration between VTE and normal samples, with the expression of DLST and LSP1 positively correlated with the content of some immune cells. The R package, code, and analysis results used in this paper are available on https://github.com/doctorlaby/my-project.

**Conclusion:** Our research is the first to utilize machine learning techniques in identifying DLST and LSP1 as significant biomarkers of VTE. With our findings, we have uncovered new insights into the underlying causes of VTE and potential treatments for affected patients.

## 1. Introduction

Venous thromboembolism (VTE) is the most prevalent thrombotic disease, affecting approximately 1 in 12 individuals over the age of 45 [1]. VTE carries significant morbidity and mortality, with 300,000 deaths attributed to VTE annually in the United States alone, making it the third leading cause of cardiovascular-related mortality [2].

VTE occurs when thrombosis develops within the veins, predominantly in the lower extremities. Disruptions in normal venous physiology, such as vascular damage, venous flow delay, or hypercoagulability, increase the likelihood of VTE. Deep vein thrombosis (DVT) in the lower limbs can migrate and result in pulmonary embolism (PE), and in severe cases, even cardiac arrest, making it the most serious complication of VTE [3].

Symptoms and clinical signs of DVT include leg pain, warmth, swelling, edema, redness, and tenderness. Indications of PE may include dyspnea, chest discomfort, syncope, hemoptysis, hypotension, and tachycardia. However, the clinical presentation of VTE is often ambiguous, making it challenging to differentiate DVT from conditions such as hematoma, cellulitis, congestive heart failure, or superficial thrombophlebitis. Similarly, the symptoms of PE can resemble those of congestive heart failure, myocardial infarction, and other medical conditions. Following the initial occurrence of VTE, patients remain at risk of recurrent thrombosis, and while anticoagulant therapy provides some protection against thrombotic recurrence, there is still a worrisome trend of VTE occurrence or recurrence, despite the use of direct oral anticoagulants for VTE prophylaxis in high-risk patients.

In summary, there is a need to explore new blood biomarkers to improve the diagnosis of VTE. Currently, D-dimer is the only reliable laboratory biomarker used in clinical settings to identify VTE and predict its recurrence. However, the specificity of high or low D-dimer levels in blood for diagnosing VTE is limited. Increased D-dimer levels may not only indicate thrombotic conditions but can also be observed in nonthrombotic diseases, including secondary fibrinolytic hyperfunction, hypercoagulable states, diffuse intravascular coagulation, renal disease, myocardial infarction, thrombolytic therapy, and neoplastic disease. Therefore, there is a substantial amount of bioinformatics data related to VTE that remains to be explored.

In this study, we combined bioinformatics and machine learning to screen for key genes associated with the diagnosis and treatment of VTE.

Bioinformatics is a discipline at the intersection of computer science and life sciences. It employs computational tools, methods, and techniques for the analysis and interpretation of biological data. With the evolution of both computer science and medicine, machine learning algorithms have become pivotal in efficiently screening and identifying significant data on a large scale. The least absolute shrinkage and selection operator (LASSO) is a regularization-based method for filtering high-dimensional data variables. Support vector machine (SVM) is a supervised machine learning method that has been widely used for model building in problems such as feature classification and avoiding overfitting by recursive feature elimination (RFE) algorithms. To find more accurate genetic features, this study used bioinformatics and 2 machine learning algorithms to screen potential genetic markers relevant to the diagnosis and treatment of VTE.

## 2. Materials and Methods

### 2.1. Screening for Differentially Expressed Genes (DEGs)

The GEO database was used to get the microarray chip GSE19151. Gene expression analysis was performed based on a platform of GPL571 (HG-U133A_2) Affymetrix Human Genome U133A 2.0 Array. The chip contained 133 blood samples, which included 70 samples from patients with VTE and 63 samples from normal controls. The criterion for detecting significant DEGs was established at an adjusted *p* < 0.05 and |log_2_ (fold change [FC])| > 0.5. For data mining and statistical studies, the R software (version 4.2.0) and the limma packages (version 3.52.2) were used. The volcano plot and heatmap were visualized using the R-packages “ggplot2” and “pheatmap.”

### 2.2. Gene Ontology (GO) Enrichment and Kyoto Encyclopedia of Genes and Genomes (KEGG) Pathway Analysis

GO encompasses three main areas: biological process (BP), cellular component (CC), and molecular function (MF). The R-language packages used for enrichment analysis and visualization of GO and KEGG pathway data from disease-related targets were “ClusterProfiler,” “org.Hs.eg.Db,” “enrichplot,” “ggplot2,” and “pathview.” A *p* value cutoff of 0.05 was set for enrichment analysis, and the outputs were applied to generate the bubble chart and bar chart.

### 2.3. Gene Set Enrichment Analysis (GSEA)

Gene functional enrichment analysis of gene sets between the two groups was carried out by GSEA. GSEA identifies group of genes that share common biological functions or regulatory profiles and exhibit coordinated expression changes, even if these changes are subtle, across a dataset. This method enables the inclusion and analysis of genes that may show only minor differences in expression but are collectively significant in their impact. The R software package “clusterprofiler” was used to perform GSEA on differential genes. The corrected *p* value cutoff was set to 0.05. The gene set “c2.cp.kegg.v7.4.symbols.gmt” was selected to serve as the reference gene set. This gene set was obtained from the Molecular Signature Database (MSigDB).

### 2.4. Selection of Hub Genes by Machine Learning

In this study, we have utilized two machine learning methods, namely, LASSO regression and SVM-RFE, in order to effectively conduct feature selection. To perform the LASSO regression analysis, we employed the “glmnet” package within the R program. The hub genes were identified by determining the optimum lambda value which resulted in the lowest classification error. We utilized the “e1071,” “kerlab,” and “caret” packages in the R software to identify the feature gene by means of SVM-RFE, via cross-validation to determine the smallest error value. We used the “venn” package within the R software to take the intersection of the analyzed genes and generate the corresponding graph.

### 2.5. Identification of Hub Genes in VTE

We used the R package “pROC” to construct the receiver operating characteristic (ROC) curves and calculated the area under curve (AUC) to assess the diagnostic value of the hub genes. AUC > 0.7 was considered to have a better diagnostic value.

### 2.6. Comparison of Immune Cell Infiltration Analysis Between Healthy and VTE Samples

The R package “GSVA” was utilized for single-sample gene set enrichment analysis (ssGSEA) to compare immune infiltration in the healthy group and VTE samples. Immune cells with a p value greater than or equal to 0.05 were considered differentially infiltrated. The correlation between hub gene expression and immune cell infiltration was further analyzed using Spearman correlation.

## 3. Results

### 3.1. DEGs of VTE

**Figure 1:**
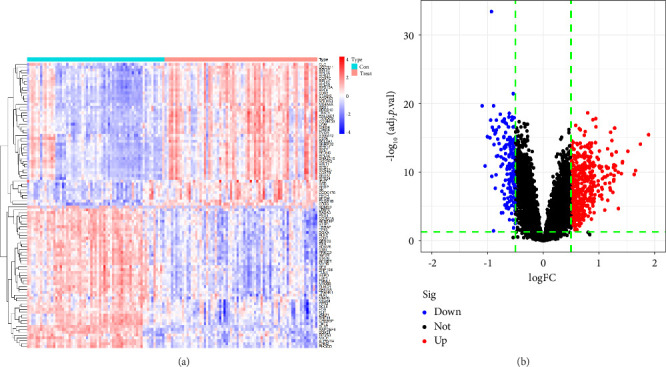
Differential expression analysis of VTE. (a) Heatmap displaying differentially expressed genes (DEGs). Samples are represented horizontally, while genes are represented vertically. High gene expression is indicated in red, and low expression in blue. (b) Volcano plot depicting DEGs between control and VTE samples. Genes significantly upregulated in VTE are marked by red dots, and downregulated genes by blue dots above the dashed lines. VTE, venous thromboembolism.

A total of 628 DEGs were screened from GSE19151 gene chip data.  Figure 1(a) shows a heatmap of these DEG expression levels, and all of them are shown graphically in Figure 1(b) as a volcanic map, with each dot standing in for a single gene.

### 3.2. GO and KEGG Enrichment Analysis

**Figure 2:**
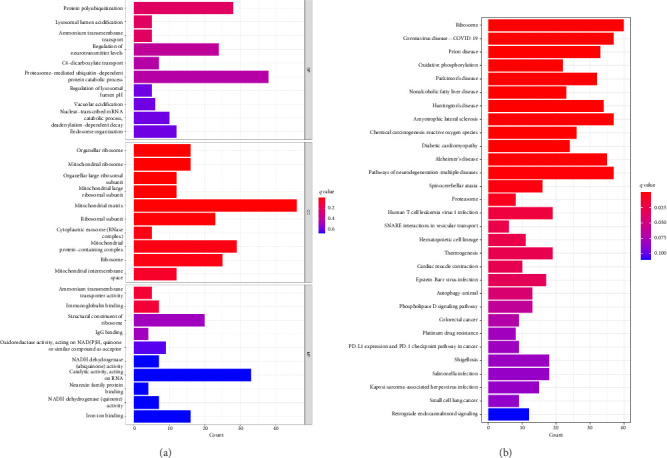
Enrichment analysis of DEGs in VTE. (a) Top 10 enriched gene ontology (GO) terms, categorized as biological process (BP), cellular component (CC), and molecular function (MF). The *x*-axis shows the number of enriched genes, and the *y*-axis shows GO terms. (b) Top 20 enriched KEGG pathways, with enriched genes on the *x*-axis and KEGG terms on the *y*-axis. KEGG, Kyoto Encyclopedia of Genes and Genomes.

We conducted GO and KEGG enrichment analyses on these DEGs, resulting in 316 GO items, including 235 BP, 39 CC, and 42 MF. The BPs identified include protein polyubiquitination, lysosomal lumen acidification, ammonium transmembrane transport, and regulation of neurotransmitter levels. The CCs identified include organellar ribosome, organellar large ribosomal subunit, mitochondrial ribosome, mitochondrial large ribosomal subunit, mitochondrial matrix, and ribosomal subunit. The MFs identified include ammonium transmembrane transporter activity, immunoglobulin binding, structural constituent of ribosome, IgG binding, and oxidoreductase activity. Furthermore, these genes were found to be significantly enriched in 51 KEGG pathways, indicating their involvement in crucial pathogenic processes such as ribosome, coronavirus disease—COVID-19, viral infection, oxidative phosphorylation, nonalcoholic fatty liver disease, Parkinson's disease, apoptosis, and cancer. The top 10 BP, CC, and MF with *p* < 0.05 are presented in Figure 2(a), and the top 30 KEGG items with *p* < 0.05 are listed in Figure 2(b).

### 3.3. GSEA

**Figure 3:**
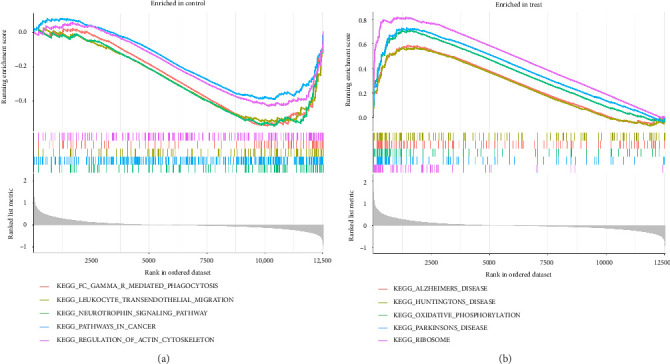
GSEA of KEGG pathways. (a) KEGG pathways significantly enriched in the control group. (b) KEGG pathways significantly enriched in the VTE group. GSEA, gene set enrichment analysis; KEGG, Kyoto Encyclopedia of Genes and Genomes.

The gene expression levels of VTE patients and healthy controls were analyzed by GSEA. The GSEA results showed a total of 44 significantly enriched gene sets. Among them, the top 5 most active KEGG pathways in the healthy group were pathways in cancer, neurotrophin signaling pathway, regulation of actin cytoskeleton, fc gamma r mediated phagocytosis, and leukocyte transendothelial migration (Figure 3(a)), while the top 5 most active KEGG pathways in the VTE group were ribosome, Parkinson's disease, oxidative phosphorylation, Alzheimer's disease, and Huntington's disease (Figure 3(b)).

### 3.4. Identification of Key Biomarkers of VTE

**Figure 4:**
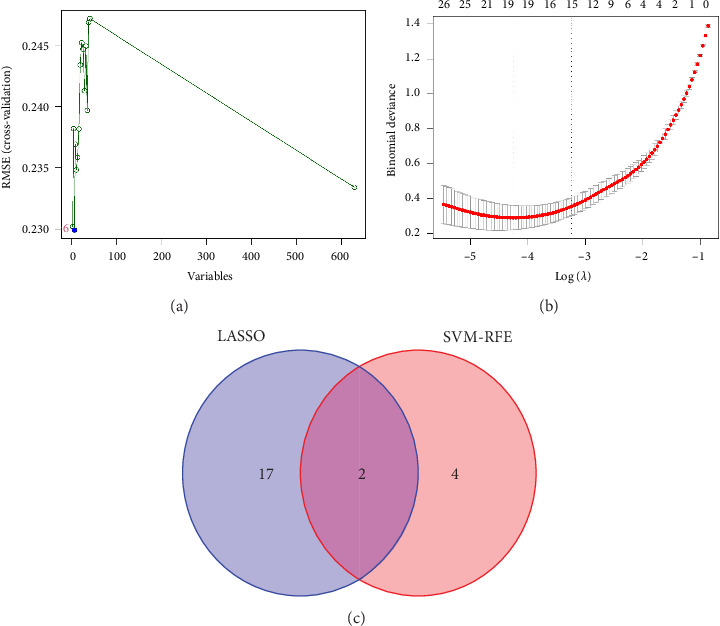
Identification of potential biomarkers for VTE diagnosis. (a) LASSO regression analysis for identifying characteristic genes of VTE. (b) Selection of optimal diagnostic biomarkers using the SVM-RFE algorithm. (c) Venn diagram showing two biomarkers identified by both SVM-RFE and LASSO methods. LASSO, least absolute shrinkage and selection operator; SVM-RFE, support vector machine-recursive feature elimination; VTE, venous thromboembolism.

In the current study, 628 DEGs were analyzed using LASSO regression analysis and SVM-RFE techniques to identify hub genes. The initial step involved importing the 628 DEGs into the LASSO regression analysis model for 10-fold cross-validation, generating 19 signature genes by selecting the point with the lowest cross-validation error (Figure 4(a)). These 19 genes were listed as follows: DLST, LSP1, RAB5C, PSMA7, RNF19B, CCDC15, RSRC1, RHOB, SNORA21, LOC100129361, CR2, SERPINE2, CD3E, RNASE6, RPL37, IFIT1, PDJK1IP1, C4BPA, and IFI44L. Next, the SVM-RFE algorithm was employed to extract six signature genes from the 628 DEGs with minimal cross-validation error (Figure 4(b)). These six genes were DLST, MYH9, LSP1, CYTH1, TRRAP, and PPP2R1A. The consequent genes obtained through both techniques were merged through a Venn analysis, culminating in DLST and LSP1 as key markers of VTE (Figure 4(c)).

### 3.5. The Predicted Value of Hub Genes

**Figure 5:**
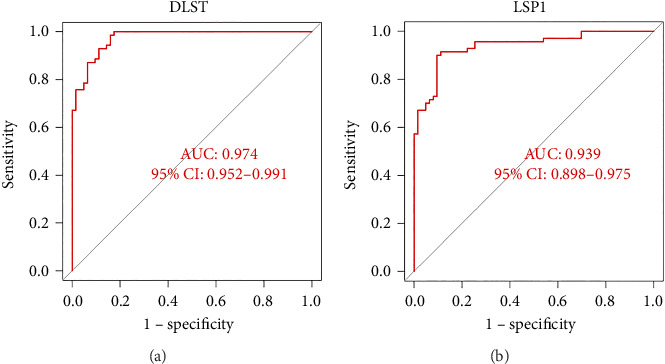
Diagnostic efficacy of biomarkers using ROC analysis. Receiver operating characteristic (ROC) analysis of hub genes DLST and LSP1, with respective area under the curve (AUC) values of 0.974 and 0.939.

ROC curve and AUC showed the predictive value of these genes as VTE biomarkers. As shown in Figure 5, the AUC values of the two core genes extracted in this study are DLST 0.974 and LSP1 0.939. The AUC values show that DLST and LSP1 are effective in predicting VTE disease.

### 3.6. Distribution of Immune Cells in VTE

**Figure 6:**
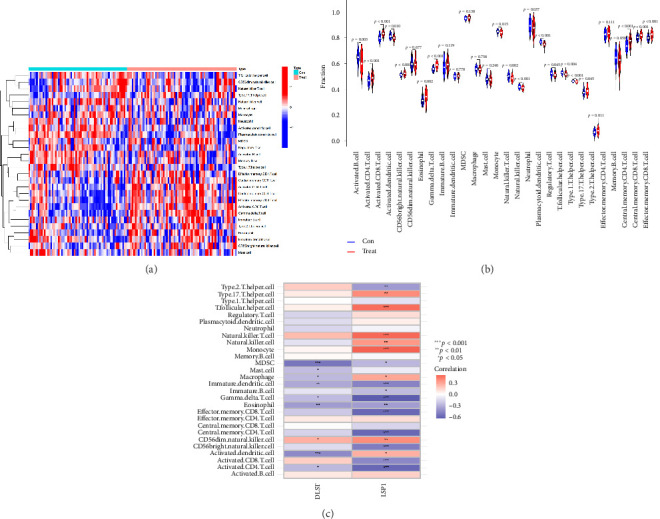
Immune cell analysis in VTE. (a) Heatmap depicting expression levels of 28 immune cell types in VTE vs. control groups. Red indicates high expression, and blue indicates low expression. (b) Violin plot illustrating differences in immune cell expression between VTE and healthy samples. (c) Correlation heatmap between immune cell infiltration and two hub genes. Positive correlations are shown in red, and negative correlations in blue.

Calculation using ssGSEA was conducted to determine the infiltration levels of 28 immune cells in VTE and normal samples, as shown in Figure 6(a). There was a significant difference in the degree of infiltration between VTE samples and healthy samples in 20 of the 28 immune cell types as demonstrated in Figure 6(b). These immune cells include activated B cell, activated CD8 T cell, activated CD4 T cell, activated dendritic cell, eosinophil, gamma delta T cell, CD56 bright natural killer cell, monocyte, natural killer cell, plasmacytoid dendritic cell, neutrophil, natural killer T cell, T follicular helper cell, regulatory T cell, type 17 T helper cell, type 1 T helper cell, type 2 T helper cell, central memory CD4 T cell, central memory CD8 T cell, and effector memory CD8 T cell. The infiltration levels of 10 immune cells in VTE samples were higher than those in healthy samples, namely, activated CD8 T cell, activated CD4 T cell, eosinophil, CD56 bright natural killer cell, gamma delta T cell, type 2 T helper cell, central memory CD4 T cell, type 17 T helper cell, central memory CD8 T cell, and effector memory CD8 T cell. We also conducted correlation analysis between immune cells and the two hub genes (Figure 6(c)). DLST exhibited a positive correlation only with CD56 dim natural killer cells. On the other hand, LSP1 expression was positively associated with monocyte, natural killer T cell, natural killer cell, CD56 dim natural killer cell, T follicular helper cell, type 17 T helper cell, macrophage, and activated dendritic cell.

## 4. Discussion

VTE is more likely to occur when there is a disturbance in normal venous physiology, such as vascular damage, venous blood flow stasis, or hypercoagulability. A blood clot can either remain stationary or dislodge into the lungs. Currently, VTE still has a high morbidity and mortality rate, affecting more than 1 million Americans and over 700,000 Europeans annually [4]. Although inpatient morbidity and mortality rates for VTE in the United States have decreased from 1999 to 2008, roughly 30% of PE patients die within the first year of diagnosis [5]. VTE is a disease affected by multiple risk factors, both hereditary and acquired; acquired factors include advanced age, surgery, active cancer, and other factors that lead to hypercoagulable states, vascular injury, or venous stasis, and hereditary factors include protein S and protein C deficiencies, mutations in the prothrombin gene, and antithrombin deficiency [6].

Venous thrombosis has a serious impact on quality of life and increases the financial burden on families, especially in the aging population. Unidentifiable factors increase the risk of recurrent VTE in individuals. If thrombosis is not diagnosed and managed in a timely manner, it can lead to long-term negative impacts on the patient's quality of life. Despite significant advances in adjuvant drug therapy and endovascular treatment strategies for VTE, it still poses a major threat to human health in cardiovascular disease. Therefore, successful screening techniques and accurate diagnosis remain a significant challenge to reducing the incidence of VTE. In this study, we successfully used gene expression profiling of whole blood samples to screen for DEGs in VTE patient samples. Bioinformatics analysis and machine learning helped us identify potential key genes in DEGs. We carried out DEG analysis and identified 628 DEGs successfully (Log2FC > 0.5 and adjusted *p* < 0.05). GO, KEGG, and GSEA enrichment analyses helped us gain more insight into the biological functions of DEGs. Subsequently, LASSO regression analysis was used to screen 19 signature genes in the DEGs, and the SVM-RFE algorithm identified 6 signature genes. The intersection of the signature genes screened by the two machine learning methods was taken to finally obtain 2 key biomarkers, DLST and LSP1, which were validated with high AUC values in ROC curves. These two key genes have good predictive value for VTE. Based on the results of ssGSEA, there was a significant difference in the degree of infiltration of most immune cells in VTE and normal samples. Furthermore, the expression of these two key genes was positively correlated with partial immune cell content.

Based on the findings of this study, the GO analysis demonstrated that the DEGs were primarily concentrated in protein polyubiquitination, lysosomal lumen acidification, organellar ribosome, mitochondrial ribosome, ammonium transmembrane transporter activity, and immunoglobulin binding. Furthermore, the results of the KEGG pathway analysis revealed that the DEGs were enriched in ribosome, coronavirus disease—COVID-19, viral infection, oxidative phosphorylation, nonalcoholic fatty liver disease, Parkinson's disease, apoptosis, and cancer. Additionally, the GSEA results indicated that the most prominent KEGG pathways in the VTE group were ribosome, Parkinson's disease, oxidative phosphorylation, Alzheimer's disease, and Huntington's disease.

The pathways of ribosome-related genes may play a crucial role in VTE. It has been reported that inflammation and thrombosis have a strong interaction effect, and now, it is believed that inflammation can be a risk factor for VTE, while inflammation is also one of the major clinical manifestations of VTE [7]. It has been demonstrated that the levels of ribosomes increase when cytokines are released at the site of inflammation [8, 9]. Furthermore, there is a growing belief that ribosomes can be targeted to address cardiovascular disease [10], which is a significant risk factor for VTE [11]. This is further evidence linking ribosomes to VTE. Further research has revealed that platelets, which act as procoagulants, can encourage the formation of nets and, in turn, DVT. Ribosomes play a crucial role in the translation of platelet proteins [12, 13]. Mitochondria also play a vital role in regulating the life and death of eukaryotic cells by mediating the conversion of aerobic energy via the oxidative phosphorylation system and controlling the internal pathways of apoptosis. Several mitochondrial ribosomal protein genes have been reported to be involved in various cellular processes, including cell proliferation, apoptosis, and the cell cycle [14, 15]. Additionally, there is a significant correlation between VTE and the processes of cell proliferation, death, and the cell cycle [16–18].

It has been reported that there is a longstanding association between the onset of blood clotting and the progression of cancer [19]. Patients with triple-negative breast cancer who exhibit high DLST expression have a poor prognosis for both their overall and disease-free survival [20]. Depletion of DLST affects the growth, survival, and migration of triple-negative breast cancer cells which retain their tricarboxylic acid cycle function but has little impact on those with a defective cycle. In DLST-dependent triple-negative breast cancer cells, glutamine contribution decreases dramatically upon depletion of DLST [20]. Additionally, DLST has been identified as a significant contributor to the aggression of neuroblastoma, highlighting oxidative phosphorylation as a major player in the development of high-risk neuroblastoma [21]. Furthermore, it has been hypothesized that acute PE induces a disconnection between the glycolysis and oxidative phosphorylation pathways within cells [22, 23].

Leukocyte-specific protein 1 (LSP1) was initially isolated from lymphocytes in 1988 and was known as LSP1. Subsequently, it has also been identified in monocytes, macrophages, endothelium, and neutrophils [24–28]. This protein is essential for the leukocyte chemotaxis process that occurs during inflammation in tissues [29]. LSP1 plays a vital role in neutrophil motility, fibrinogen matrix protein adhesion, and migration through endothelial layers [30–32]. A deficiency of LSP1 is likely to disrupt the fundamental inflammatory process, including the recruitment of neutrophils and eosinophils, and the associated cytokine network to reduce inflammation [29, 32]. During the inflammatory process, endothelial LSP1 integrates into the cytoskeleton and contributes significantly to the formation of the endothelium dome, regulating the migration of neutrophils from vascular endothelial cells [33–36].

## 5. Conclusion

In summary, since there are currently no reliable disease-specific predictive markers for VTE, we conducted a bioinformatics analysis based on blood expression profiles associated with VTE. Our findings suggest that the risk of developing VTE can potentially be determined by testing for two hub genes, DLST and LSP1. Moreover, our study offers valuable information and ideas for further research of the functions of DLST and LSP1 in VTE by describing the infiltration pattern of immune cells in the condition. Ultimately, our study provides unique insights into the underlying processes of VTE and serves as a valuable data reference for future clinical investigations.

## Data Availability

The data used to support the conclusions of this research are available on https://github.com/doctorlaby/my-project.
